# Effectiveness of a decision aid for promoting colorectal cancer screening in Spain: a randomized trial

**DOI:** 10.1186/s12911-019-0739-6

**Published:** 2019-01-10

**Authors:** Lilisbeth Perestelo-Perez, Amado Rivero-Santana, Alezandra Torres-Castaño, Vanesa Ramos-Garcia, Yolanda Alvarez-Perez, Nerea Gonzalez-Hernandez, Andrea Buron, Michael Pignone, Pedro Serrano-Aguilar

**Affiliations:** 1 Evaluation Unit of the Canary Islands Health Service (SESCS), s/n. 38109. El Rosario. S/C de Tenerife, Tenerife, Spain; 2 Health Services Research on Chronic Patients Network (REDISSEC), Tenerife, Spain; 3 Center for Biomedical Research of the Canary Islands (CIBICAN), Tenerife, Spain; 4 Canary Islands Foundation of Health Research (FUNCANIS), Tenerife, Spain; 5 Research Unit. Hospital Galdakao-Usansolo, Bilbao, Bizkaia Spain; 6 Epidemiology and Evaluation Unit. Hospital del Mar, Barcelona, Spain; 7 Dell Medical School. University of Texas, Austin, TX USA

**Keywords:** Colorectal cancer screening, Decision aid, Patient involvement, Primary care, Randomized controlled trial, Shared decision-making, Spain

## Abstract

**Background:**

Colorectal cancer (CRC) screening has shown to reduce incidence and mortality rates, and therefore is widely recommended for people above 50 years-old. However, despite the implementation of population-based screening programs in several countries, uptake rates are still low. Decision aids (DAs) may help patients to make informed decisions about CRC screening.

**Methods:**

We performed a randomized controlled trial to assess the effectiveness of a DA developed to promote CRC screening, with patients from two primary care centers in Spain who never had underwent CRC screening. Contrary to center B (*n* = 24), Center A (*n* = 83) attended patients from an area where the population-based screening program was not implemented at that moment. Outcome measures were decisional conflict, knowledge of the disease and available screening options, intention to uptake the test, and concordance between patients’ goals/concerns and intention.

**Results:**

In center A, there were significant differences favoring the DA in decisional conflict (*p* < 0.001) and knowledge (*p* < 0.001). The absolute differences favoring DA group in intention to undergo fecal occult blood test (10.5%) and colonoscopy (13.7%) were significant only before correction for attenuation. In center B the differences were significant only for knowledge (*p* < 0.001). Patients’ goals and concerns regarding the screening did not significantly predict their intention, and therefore we could not calculate a measure of concordance between the two constructs.

**Conclusions:**

A DA improved the decisional process of participants who had never been invited to participate in the Spanish public CRC screening program, replicating previous results in this field. Future research is needed to identify subgroups that could benefit more from these interventions.

**Trial registration:**

International Standard Registered Clinical/social Study Number: ISRCTN98108615 (Retrospectively registered on 27 December 2018).

## Background

Colorectal cancer (CRC) is the third most common type of cancer in the world and one of the main causes of death by cancer, showing a wide geographical variation in incidence and mortality [1]. In western countries, these rates have declined from several decades ago due to the introduction of screening techniques (e.g., fecal occult blood test -FOBT-, colonoscopy, sigmoidoscopy), and the improvement in treatment [2–4]. CRC screening is therefore recommended for people above 50-years old, and population programs have been implemented in several countries at a national or regional level, however screening rates are still low [5–7]. Research has shown that screening uptake is significantly influenced by a wide range of sociodemographic, clinical, psychosocial, lifestyle and health system-related variables [8–11].

Proposed interventions to increase and improve recruitment for CRC screening have included reminders by health professionals, informational material in different formats (printed, videos, interactive computer programs), mailed FOBT kits, group education, counseling or provider or practice-directed interventions [12–14]. One of these practice-based interventions is the application of decision aids (DA). These tools include explanations of the available treatment or screening/diagnostic procedures, evidence-based quantitative information about their benefits and risks, and promote implicit or explicit clarification of patients’ preferences about testing and its potential consequences [15, 16].

DAs have shown to improve patients’ decisional process (e.g., knowledge of the disease and treatments, risk perceptions, decisional conflict, congruence between preferences and choices) in many physical and mental health conditions [17]. In the field of CRC, Volk et al. [18] recently published a systematic review and meta-analysis on the effectiveness of DAs. Pooled results showed that, compared to no-intervention, they significantly increased participants’ knowledge, and screening intention and uptake, but when control groups were given general CRC screening information, only the difference in knowledge was significant.

In Spain, public population-based CRC screening programs have been heterogeneously implemented across and within its 17 autonomous regions, since the first initiative launched in Catalonia in 2000 [19]. Programs follow the European Guidelines, are entirely free of charge and biennially invite men and women aged 50 to 69 years to a FOBT test; colonoscopy as a diagnostic test is offered to those FOBT positive. Although screening rates have progressively increased through the successive rounds of the program, reaching about 40% of eligible population, they still are below the optimal recommended value or standards [20].

In this context, the aim of this study is to assess the effectiveness of a DA on the basic decisional processes (knowledge, decisional conflict, intention) about CRC screening of Spanish primary care patients eligible for the procedure. Secondarily, since in our region (Canary Islands) the public screening program has been implemented only in some areas, we wanted to assess the effect of the DA in participants who previously had been invited (and refused) to be screened compared to those who had never been invited.

## Methods

We performed a randomized controlled trial (RCT) to assess the effectiveness of a web-based DA developed to promote informed decisions about CRC screening. Inclusion criteria were: age 50–69 years-old, having no CRC history or current symptoms, no family history of CRC, and no previous CRC screening. Two primary care centers in Tenerife (Canary Islands, Spain) participated in the study, carried out during 2016. One of them (hereafter, center A) attends patients in an area where the public CRC screening program was not implemented at that moment, although it was expected to be accessible in the next years. The other (center B) is in an area where the screening program have been running since 2009, and therefore its patients between 50 and 69 years-old had received invitations and reminders in the past to undergo the screening (but not specific information about the procedure). Collaborating physicians in both centers assessed the eligibility of their consecutive patients, and invited those eligible to be contacted by telephone by a member of our team, who explained the study in more detail and established an appointment in their primary care center.

Computer-based simple randomization was performed by a statistician not involved in the study, and the researcher who recruited participants and established an appointment by phone was blinded to allocation (we used a centralized off-site computer allocation process). All participants were received in a room of their primary care center by other researcher, who had the allocation list; all participants were assigned to the group in which they had been randomized. Intervention participants signed informed consent and reviewed the DA in the computer; the researcher was sitting beside them and briefly explained them the functioning of the DA and gave support in navigation when necessary. After that, participants filled the questionnaires assessing the outcome variables, in the same web-based interface, with no mediation of the researcher. Control participants only signed informed consent and completed the questionnaires, in a different interface of the web site. Therefore, all measures were assessed only once (after reviewing the DA in the intervention group), except knowledge in the DA group, which was assessed both before and after the DA application.

### Decision aid

The DA was a Spanish translation and adaptation of the one developed by Pignone et al. [21]. The adaptation process was performed with the support of an advisory group of health professionals related to CRC care. We carried out four focus groups with professionals and citizens eligible for screening (who had undergone the procedure or not), before and during the adaptation process. The final DA is presented in a web format that presents written information based on scientific evidence about CRC (available in: www.pydesalud.com/toma-de-decisiones-en-cancer-colorrectal/). It is organized in three sections; the first one explains the usefulness of DAs and a brief summary of their content. The second section explains CRC causes, symptoms and available treatments. Third, information on FOBT and colonoscopy is presented, including quantitative data about incidence and mortality risk reduction, as well as potential adverse effects. Finally, a summary table is presented with the two tests and their characteristics (i.e., description, preparation, recovery, frequency, need for additional tests, incidence and mortality risk reduction, adverse effects) compared to not being screened. Outcomes variables are then assessed in the same interface. Once the questionnaires have been completed, a summary document including the content explored, together with the answers given by the patients is automatically generated and available for them, in a printed format or via e-mail.

### Measures

The primary outcome measure was decisional conflict, defined as uncertainty about the course of action to take when choosing among several medical procedures [21]. It was measured with the Spanish version of the Decisional Conflict Scale (DCS) [22, 23]. It includes 16 items and 5 subscales (with 3 items each, except the latter, with 4): feeling informed, having clear values about benefits and risks, support to take the decision, uncertainty, and perceived effectiveness of the decision. Items are scored from 0 (strongly agree) to 4 points (strongly disagree), with higher scores indicating higher decisional conflict. Scores on the total scale and subscales are transformed to a 0–100 scale. In two previous trials with Spanish patients diagnosed with type-2 diabetes and depression, respectively, we observed high internal consistency values (Cronbach alpha of 0.90 and 0.88) [24, 25]. We calculated that, assuming equal variances in the intervention and control groups, 126 participants would be needed to detect a moderate effect size (standardized mean difference = 0.5), with a confidence level of 95% and a power of 80%.

### Secondary outcomes

Knowledge of colorectal cancer and screening options was assessed using a measure based on preliminary studies testing a Spanish-Language CRC screening decision aid in Latinos with limited English proficiency [26]. It includes 12 items with response format of “true/false/I don’t know”. The percentage of correct responses represents the knowledge score. The content of all items was explained in the DA.

We could not assess actual screening uptake in center B, and participants of center A did not have access to the public screening program and therefore could only undergo screening by means of private services, but this was considered improbable given the out of pocket costs. Therefore, we instead used intention to be screened as a proxy, which has shown to be a good predictor of CRC screening uptake [27, 28]. Patients were asked two questions about their intention to undergo FOBT and colonoscopy, respectively (yes/no). Finally, we assessed the attributed importance for eight characteristics of screening when making a decision about it (goals/concerns), rating them from 1 (not important at all) to 5 (extremely important).

### Statistical analyses

We performed one 2 × 2 analysis of variance (ANOVA) with each of the continuous outcomes (DCS and its subscales, knowledge) as dependent variable, and the intervention group (DA vs. control) and center (A/B) as independent variables. The change in knowledge in the DA group was analyzed by means of paired t-test. To assess differences between the DA and control groups in the intention to undergo FOBT and/or colonoscopy, respectively, we performed χ^2^ analyses for the whole sample and separately by center; since several of these analyses yielded expected frequencies < 5 in the “no screening” cells, we also report the results corrected for continuity (Yates’ correction).

The concordance between patients’ goals/concerns about the screening procedure and their intention to be screened was calculated as proposed by Sepucha et al. [29] for operationalizing concordant decisions. First, univariate logistic regressions were performed with intention as dependent variable and each of the goals/concerns about the screening procedure rated by patients as predictor. Those that showed a significant association were introduced together in a multiple logistic regression analysis, again with the intention as dependent variable. After excluding those that were no longer significant, the final model is used to calculate each subject’s predicted probability of stating an intention to be (probability ≥0.5) or not to be (< 0.5) screened. Finally, participants were classified as concordant if their predicted probability coincided with their stated intention, and vice versa. Differences between DA and control groups in the rate of patients with concordant intentions was calculated by means of a χ^2^ test.

## Results

**Fig. 1:**
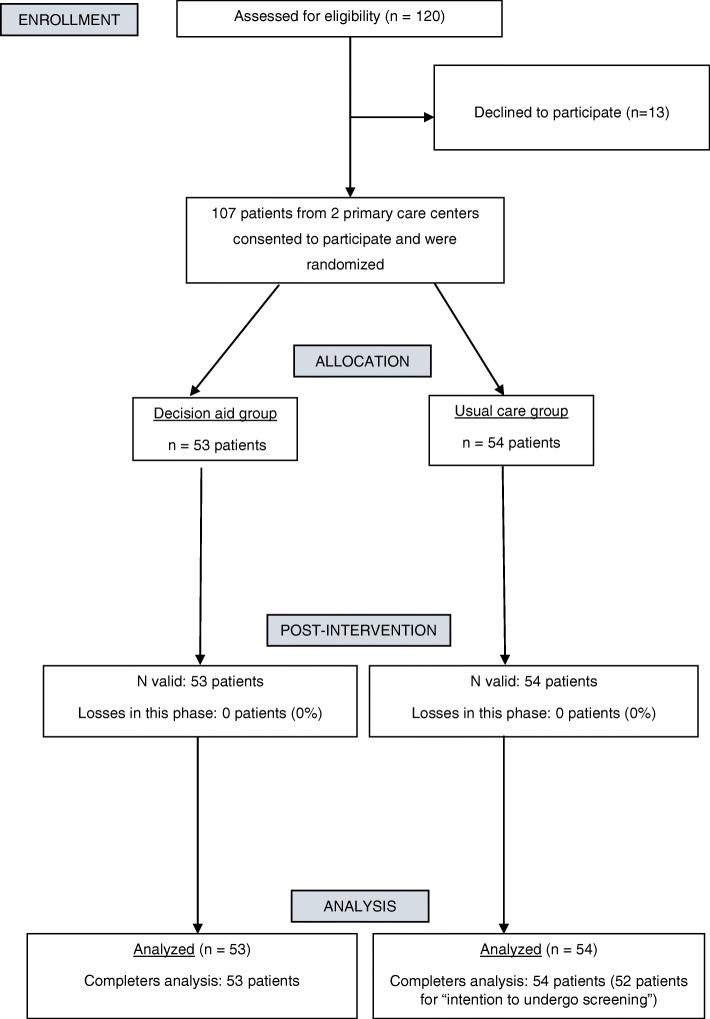
CONSORT flow diagram of participants through the study

Figure 1 shows the participants’ flow chart and Table 1 their baseline characteristics. One hundred and twenty patients were informed about the study by the recruiter researcher, from whom 13 decline participation. Therefore, 107 participants were included, 83 from center A and 24 from center B, and 53 and 54 patients were randomized to intervention (DA) and control groups, respectively. Mean age was 58.1 years-old, 57.9% were female and a 45.7% had completed secondary education. Patients from center A were significantly older than those from center B (mean of 59 vs. 55 years, respectively, *p* = 0.004) and included fewer women (52% vs.79%, *p* = 0.017).

**Table 1:** Baseline participants’ characteristics

	DA group (*n* = 53)	Control group (*n* = 54)
Age	57.9 (5.93)	58.4 (6.30)
Female	33 (62.3)	29 (53.7)
Education		
No primary studies	7 (13.2)	12 (22.6)
Primary studies	17 (32.1)	21 (39.6)
Secondary studies	21 (39.6)	15 (28.3)
Universitary studies	8 (15.1)	5 (9.4)
Center A/B	43/10 (81%/19%)	40/14 (74%/26%)

*DA* decision aid

Table 2 shows the results of the different ANOVA performed for each of the continuous outcomes measures. The effect of center was significant only for the “informed” subscale, with patients from center B showing significantly less conflict (*p* < 0.001).

**Table 2:** Effect of the intervention on knowledge and decisional conflict^a^

	Center A		Center B		DA effect	Center effect	Interaction
	DA (*n* = 43)	Control (*n* = 40)	DA (*n* = 10)	Control (*n* = 14)			
Knowledge	75 (14.4)	59.4 (14.4)	87.5 (11.3)	60.1 (17.4)	39.6 (< 0.001)	3.83 (0.055)	2.96 (0.088)
DCS	22.2 (14.6)	48.9 (21.3)	29.7 (11.8)	29.2 (12.2)	10.8 (0.001)	2.31 (0.131)	11.5 (0.001)
Informed	23.1 (17.5)	85.0 (20.6)	33.3 (20.8)	33.9 (17.1)	49.3 (< 0.001)	21.0 (< 0.001)	47.5 (< 0.001)
Values	19.0 (14.7)	45.2 (39.0)	31.7 (11.0)	30.4 (14.8)	4.02 (0.048)	0.03 (0.861)	4.90 (0.029)
Support	21.3 (17.4)	29.2 (18.5)	25.8 (9.17)	29.8 (13.4)	2.24 (0.138)	0.42 (0.518)	0.25 (0.620)
Uncertainty	25.4 (18.9)	50.2 (34.7)	32.5 (19.8)	27.4 (22.3)	2.53 (0.115)	1.61 (0.208)	5.84 (0.017)
Effectiveness	22.2 (17.1)	38.3 (29.8)	26.3 (7.68)	25.9 (14.9)	2.29 (0.133)	0.65 (0.421)	2.51 (0.116)

^a^Means (sd) by group and center, and results of ANOVAs (F, p-value). For knowledge (range 0–100), higher scores indicate a better result, and the opposite applies for the decisional conflict scale (DCS) and its subscales (range 0–100). *DA* decision aid

Regarding the effect of the intervention on decisional conflict, the interaction term was significant for the total score and three subscales (informed, values and uncertainty); post-hoc analyses showed that the DA only was effective in center A (*p* < 0.001). For the other two subscales there were not significant main or interaction effects, but when analyzed separately in center A, t-tests yielded *p*-values of 0.003 (effectiveness) and 0.05 (support) favoring the DA. Differences ranged from 7.2 (support) to 62 points (informed).

Knowledge in the control group was not significantly different from pre-intervention scores in the DA group (*p* = 0.714), which after the intervention improved 16.6 points on average (*p* < 0.001). ANOVA yielded a significant main effect of the DA on knowledge (mean difference: 17.8%, *p* < 0.001).

In the whole sample, most participants stated an intention to undergo FOBT (92.4%) or colonoscopy (89.5%). In center A, the differences favoring the DA (FOBT: 100% vs. 89.5%; Colonoscopy: 95.3% vs. 81.6%) were significant for both procedures in the uncorrected analysis, but not when Yates’ correction was applied (Table 3). In center B, none of the analyses yielded significant results.

**Table 3:** Intention to undergo colorectal cancer screening

Intention	FOBT				Colonoscopy			
	Center A (*n* = 81*)		Center B (*n* = 24)		Center A (*n* = 81*)		Center B (*n* = 24)	
	DA	Control	DA	Control	DA	Control	DA	Control
Yes	43 (100%)	34 (89.5%)	9 (90%)	11 (78.6%)	41 (95.3%)	31 (81.6%)	9 (90%)	13 (92.9%)
No	0 (0%)	4 (10.5%)	1 (10%)	3 (21.4%)	2 (4.7%)	7 (18.4%)	1 (10%)	1 (7.1%)
χ^2^ (p-value)	4.76 (0.029)		0.55 (0.459)		3.87 (0.049)		0.06 (0.803)	
χ^2^ corrected for continuity	2.78 (0.095)		0.34 (0.853)		2.60 (0.107)		0.00 (1.000)	

*Two missing values in the control group; *FOBT* fecal occult blood test, *DA* decision aid

Table 4 shows the results obtained for each of the goals/concern evaluated. Reducing both the probability of developing colorectal cancer (78% of patients scoring 5 in a 1–5 scale) and dying by its cause (91%) were rated by participants as the most important issues when deciding about CRC screening, followed by the risk of complications (70%). Univariate associations between the importance attributed to each goal/concern and the intention to get screened were assessed by means of logistic regression and no significant results were observed. Including center as covariate or using non-parametric analyses (Mann-Whitney’s U, Kolmogorov-Smirnov’s test) did not change the results; therefore, a measure of concordance between goals/concerns and intention to be screened could not be constructed.

**Table 4:** Participants’ goals and concerns about the screening procedure. Values are percentages, except for the right column (mean, sd)

	1 (not important at all)	2	3	4	5 (extremely important)	Mean (sd)
Place in which the test is performed	11.2	9.3	14.0	23.4	42.1	3.76 (1.38)
Preparation process	15.9	15.0	13.1	21.5	34.6	3.44 (1.49)
Recovery process	14.0	14.0	15.9	20.6	35.5	3.50 (1.45)
Frequency of the test	16.8	12.1	10.3	24.3	36.4	3.51 (1.50)
Risk of complications	2.8	0.9	3.7	22.4	70.1	4.56 (0.85)
Possibility of needing additional tests	8.4	9.3	9.3	31.8	41.1	3.88 (1.28)
Reducing the probability of developing colorectal cancer	0.9	0	0.9	20.6	77.6	4.74 (0.57)
Reducing the probability of dying due to colorectal cancer	0	0	0	9.3	90.7	4.91 (0.29)

## Discussion

This study aimed to assess the effectiveness of a DA for CRC screening on decisional conflict and knowledge of targeted Spanish participants who had never been screened, recruited in primary care services. We also evaluated the participants’ intention to undergo the screening procedure. In addition, we included a small subsample of participants (center B) who had previously been invited (and refused) to be screened in the public program.

In center A, results confirmed the beneficial effect of the DAs on the patients’ decisional process found in previous studies in CRC [18] and other diseases [17]. Intervention group showed significantly higher knowledge (pre-post difference of 17 points) and lower decisional conflict than control participants, with a strong effect in the DCS Informed subscale (between-group difference of 62 points). Control participants showed an acceptable level of knowledge (60% of correct responses), and nonetheless they expressed a high level of feeling uninformed. This result is in line with the relatively low correlation between objective and subjective knowledge usually observed in previous research [30–32] (*r* = 0.35 in this study), and highlights the need of educating about health topics not only to empower citizens with poor knowledge about these issues, but also to improve the subjective perception of been informed in those who already have a correct level of knowledge.

In center B, the DA also significantly improved participants’ knowledge, but had no effect on decisional conflict. Although the statistical power of the analysis was very low due to the small sample size, differences were minimal for the DCS total score and its subscales. A possible floor effect could be present, since the control group obtained relatively low scores (29 points in the total score). The effect of center was significant only for the subscale informed, with less conflict in center B. This could be reflecting the effect of the public program’s invitations and reminders provided to these participants in the previous years, although this must be interpreted cautiously due the small sample size and the differences observed between centers in age and gender composition.

The percentage of participants who stated an intention to undergo screening was very high in the whole sample. Control group obtained a rate around 85%, a value markedly greater than the observed in previous trials with control groups without intervention [33, 34], or even in those in which control participants were given general information about CRC [35–37]. Although intention significantly predicts screening uptake [26, 27], this seems to be an “inflated” result since screening rates in our region are around 40% [20]. A possible explanation is that we used a yes/no response format (whether the patient had intention to be screened or not) but not the “intensity” of intention (usually assessed with a Likert scale [37–39]), nor did we make explicit a time frame in which the decision had to be made. This could make it to seem more “hypothetical” for participants (and maybe more prone to desirability bias), and lead those in a more contemplative stage of decision making, in terms of the Transtheoretical Model of behavior change [40], to answer affirmatively. Performing the study physically in a health context (primary care centers) could also increase this desirability bias. In center A, the effect of the intervention was significant only before applying correction for continuity; nonetheless, the study was underpowered to detect the between-group difference estimated in the meta-analysis of Volk et al. [18]: 66% in the DA groups versus 45% in control groups with no intervention (168 participants would have been needed, with a 95% confidence level, to obtain a statistical power of 80%), and therefore we cannot draw definitive conclusions about the effect of the DA on this measure. Finally, we did not find any significant associations between goals/concerns and intention, a result that could be influenced by the low variability observed for both measures.

There are several limitations in this study, beyond the absence of screening uptake assessment. We did not assess decisional conflict and intention at baseline, although randomization is expected to prevent biases in this sense. The absence of intervention in the control group may introduce a “novelty effect” in favor of the DA; to minimize as much as possible this potential bias we tried to offer the same experience (except for the application of the DA) to intervention and control participants: appointment in their primary care center, standardized instructions provided by the researcher, who did not mediated in participants’ responses to questionnaires, which were delivered in the same way. Regarding external validity, a selection bias could be present since we recruited participants in primary care centers, who might not be completely representative of the population targeted for CRC screening. With these limitations in mind, the results confirmed the improvements of participants’ knowledge observed in previous studies, and add evidence about the DAs effect on decisional conflict (a measure little used previously in the field of CRC screening), supporting their effectiveness to promote informed decisions about CRC screening. We also have provided preliminary evidence on the null effect of the DA on decisional conflict in participants who have been invited (and refused) to participate in a public screening program.

Beyond the positive effects of DAs effects on cognitive outcomes, however, their effect on intention and screening uptake are far from the desired rates from a public health perspective. The meta-analysis of Volk et al. [18] shows that the pooled rate in any DA group for screening intention/interest was 66% (95% CI: 54–77%) and only 33% (95% CI: 22–46%) for screening uptake. These results highlight the necessity to include a motivational component in interventions aimed to improved screening rates. On the other side, DAs outperform general information materials at improving knowledge, but not at increasing the rate of CRC screening, which raises questions about the quantity and specificity of information necessary or sufficient to decide to undergo the test.

## Conclusions

Decision aids represent a useful resource to improve the quality of the decisional process about colorectal cancer screening, in terms of higher knowledge and less decisional conflict. However, preliminary evidence suggests that the effect on decisional conflict is not significant in people who have been previously invited to participate in a public screening program (although not specifically informed about the procedure). Future research should identify what subgroups could benefit more from DAs, as could occur with vulnerable populations [41, 42], and explore how values and motivational factors interact with knowledge and cognitive processes when making the decision about screening.

## Abbreviations

ANOVA: Analysis of variance
CRC: Colorectal cancer
DA: Decision aid
DCS: Decisional Conflict Scale
FOBT: Fecal occult blood test
RCT: Randomized controlled trial
